# In Vitro Activity of Silver-Bound Titanium Dioxide (Tiab) Against Multidrug-Resistant Bacteria from Dermatological Infections

**DOI:** 10.3390/diseases13090277

**Published:** 2025-08-22

**Authors:** Lorenzo Drago, Fabiana Giarritiello, Loredana Deflorio, Angela Uslenghi, Vincenzo Minasi, Matteo Covi, Luigi Regenburgh De La Motte

**Affiliations:** 1 UOC Laboratory of Clinical Medicine with Specialized Areas, IRCCS MultiMedica, Via G.Fantoli 16/15, 20128 Milan, Italy; lorenzo.drago@unimi.it (L.D.); f.giarritiello@studenti.unimol.it (F.G.); loredana.deflorio@multimedica.it (L.D.); angela.uslenghi@multimedica.it (A.U.); vincenzo.minasi@multimedica.it (V.M.); matteo.covi@multimedica.it (M.C.); 2 Clinical Microbiology and Microbiome Laboratory, Department of Biomedical Sciences for Health, University of Milan, 20133 Milan, Italy; 3 Department of Medicine and Health Sciences “V. Tiberio”, University of Molise, 86100 Campobasso, Italy

**Keywords:** TiAB, silver ions, dermatology, skin infections, multidrug-resistant bacteria, topical antimicrobial agent

## Abstract

Objectives: To evaluate the in vitro antimicrobial activity of TiAB, a compound based on silver-bound titanium dioxide, against clinical isolates from dermatological infections. Methods: We tested 155 strains clinically isolated from ulcers and skin infections, including MRSA, ESBL-producing *Enterobacterales*, and *P. aeruginosa*. MIC and MBC values were determined using broth microdilution according to CLSI guidelines. Time-kill assays were performed at 0.5×, 1×, and 2× MIC. Median values were used to describe susceptibility profiles. Results: TiAB exhibited strong bactericidal activity against Gram-negative bacteria, including ESBL-positive *E. coli* and *K. pneumoniae*, with complete killing at 2× MIC (4–8%) within 4–8 h. Gram-positive pathogens exhibited higher MICs (≥8%) and limited response within 24 h; however, extending exposure to 48 h resulted in enhanced activity. Conclusions: TiAB exhibited in vitro bactericidal activity with median MIC values ranging from 1% to 2% (*w*/*v*) against Gram-negative clinical isolates such as *E. coli* and *P. aeruginosa*, and 2% to 4% against Gram-positive strains including MRSA. Time-kill assays confirmed ≥3 log_10_ CFU/mL reductions for Gram-negative bacteria at 2× MIC within 24 h. These results suggest TiAB’s potential as a topical antimicrobial agent, though further in vivo studies are needed to validate its safety and efficacy.

## 1. Introduction

Skin and soft tissue infections (SSTIs), including ulcers, pressure injuries, and chronic wounds, represent a substantial clinical and economic burden in both outpatient and inpatient care, especially among immunocompromised, diabetic, and elderly patients [1]. These lesions are frequently colonized by polymicrobial communities that form resilient biofilms, which significantly delay wound healing, shield bacteria from host immune responses, and increase bacterial tolerance to conventional antimicrobials [2,3]. The most common pathogens in SSTIs include Gram-positive cocci such as *Staphylococcus aureus*—particularly methicillin-resistant *S. aureus* (MRSA)—and *Streptococcus pyogenes*, as well as Gram-negative bacilli like *Pseudomonas aeruginosa*, *Escherichia coli*, and *Klebsiella pneumoniae* [4,5,6]. *P. aeruginosa* is especially problematic due to its ability to persist in moist wound environments and its high intrinsic and acquired resistance mechanisms, including efflux pumps, porin loss, and robust biofilm formation [7]. The increasing prevalence of multidrug-resistant (MDR) strains, including extended-spectrum β-lactamase (ESBL)-producing Enterobacterales and carbapenem-resistant *P. aeruginosa*, presents a major obstacle to treatment [8]. In this context, there is renewed interest in topical antimicrobial strategies to help reduce local bacterial load and minimize the selection pressure associated with systemic antibiotics [9]. Among topical antimicrobials, inorganic nanomaterials such as silver nanoparticles (AgNPs), zinc oxide (ZnO), and titanium dioxide (TiO_2_) have demonstrated broad-spectrum activity against bacteria and fungi, with applications in wound care and medical device coatings [10,11]. However, conventional silver-based products—for example, silver sulfadiazine creams and silver hydrogel dressings—can have drawbacks, including rapid ion release, short duration of effect, and potential cytotoxicity to host tissues [12]. Recent research has therefore focused on advanced nanostructures that allow better control over silver ion release, enhanced stability, and improved safety profiles [13]. TiAB is a novel hybrid nanomaterial in which silver ions (Ag^+^) are covalently bound to microcrystalline TiO_2_, enabling a more stable and sustained release of bioactive silver while reducing environmental accumulation and potential toxicity [14]. This distinguishes TiAB from traditional AgNPs, which can aggregate or release silver ions unpredictably [15]. Recent studies on Ag–TiO_2_ nanocomposites have shown promising antibacterial and antibiofilm performance against MDR strains, along with improved biocompatibility in vitro [16,17,18]. Moreover, a recent clinical trial demonstrated the benefits of AgNP formulations in enhancing wound healing and bacterial clearance in infected wounds [19], supporting the potential clinical relevance of novel silver-based compounds. For example, Puca et al. demonstrated the effectiveness of a TiAB-based medical device in reducing biofilm formation by *S. aureus*, *Enterococcus faecalis*, and *P. aeruginosa* on surgical sutures [20]. However, existing studies have generally been limited to reference strains or specific laboratory models, and there is still a lack of robust data on TiAB’s activity against a diverse panel of clinical isolates from real-world SSTIs. Therefore, the present study aims to address this knowledge gap by comprehensively evaluating the in vitro antimicrobial activity of TiAB against 155 clinical isolates obtained from patients with SSTIs, including both Gram-positive and Gram-negative pathogens. By conducting MIC, MBC, and time-kill assays, this work seeks to better characterize TiAB’s antimicrobial profile and assess its potential suitability for topical use in dermatological practice, particularly in an era of escalating antimicrobial resistance.

## 2. Materials and Methods

### 2.1. Bacterial Strains

This study evaluated 155 clinical isolates obtained from patients with skin infections, ulcers, and pressure sores. The isolates were collected, cryopreserved at −80 °C using the bead technique, and stored in the strain library of MultiMedica IRCCS Laboratory (Milan, Italy). The tested strains included: *Streptococcus pyogenes* (*n* = 15); *Staphylococcus aureus* methicillin-sensitive (MSSA, *n* = 15); *Staphylococcus aureus* methicillin-resistant (MRSA, *n* = 15); *Staphylococcus epidermidis* methicillin-sensitive (MSSE, *n* = 10); *Staphylococcus epidermidis* methicillin-resistant (MRSE, *n* = 10); *Escherichia coli* (*n* = 15); *Escherichia coli* extended-spectrum beta-lactamase (ESBL)-positive (*n* = 15); *Enterobacter cloacae* (*n* = 15); *Klebsiella pneumoniae* (*n* = 15); *Enterococcus* spp. (*n* = 15); and *Pseudomonas aeruginosa* (*n* = 15). Prior to testing, strains were thawed and cultured on Columbia Agar with 5% Sheep Blood (COS agar, cod. 43041, bioMérieux italia S.p.A, Firenze, Italy), then incubated at 37 °C under aerobic conditions for 24 h to confirm viability. A 0.5 McFarland suspension (approximately 1.5 × 10^8^ CFU/mL) was prepared from each viable colony in Brain Heart Infusion broth (BHI, cod. 42081, bioMérieux) using a DensiCHEK Plus spectrophotometer (bioMérieux) for subsequent MIC and MBC testing. Antimicrobial susceptibility was determined using the automated VITEK system (bioMérieux) following the manufacturer’s instructions and CLSI guidelines. Methicillin-resistant *Staphylococcus aureus* (MRSA) strains were additionally screened using selective chromogenic agar plates specific for MRSA detection.

### 2.2. Preparation of TiAB Suspension

The TiAB powder was provided by Eurokemical S.r.l. (Covo, Italy) lot.01/22. The compound consisted of 96.00% to 99.4% of titanium dioxide (TiO_2_) and silver ions at a concentration range of 0.18–0.23%, with a pH of 3.5 in a 2% aqueous solution. Moisture content was 0.1%, and the total microbial count was <100 CFU/g, confirming the sterility of the product. The powder was stored at room temperature under sterile conditions until use. For experimental procedures, a TiAB suspension was prepared at an initial concentration of 16% by dissolving 2 g of powder in 12.5 mL of Brain Heart Infusion (BHI) broth. To ensure homogeneity and prevent sedimentation, the suspension was continuously vortexed and maintained under agitation throughout testing. Serial 1:2 dilutions were prepared in 96-well microplates, resulting in final concentrations ranging from 8% to 0.01%. Each first well contained 100 µL of TiAB at 8%, 100 µL of BHI broth, and 100 µL of bacterial suspension adjusted to 0.5 McFarland.

### 2.3. Media and Culture Conditions

Brain Heart Infusion (BHI) broth was selected for MIC and MBC assays due to its enriched nutrient composition, which supports robust growth of diverse clinical isolates from dermatological infections. Preliminary experiments demonstrated that BHI provided more consistent and reproducible growth results with TiAB compared to Mueller-Hinton Broth, which is traditionally used but showed variability in this context. Columbia Agar with 5% Sheep Blood (COS agar) was employed for MBC determinations, as it is well-suited for fastidious organisms frequently isolated in skin infections, enabling clear visualization and reliable enumeration of surviving colonies. It should be noted that TiAB exhibits limited solubility in liquid media, leading to partial sedimentation and potential interference with optical turbidity assessments. Consequently, MIC endpoints were primarily determined via visual inspection of turbidity combined with confirmatory colony counts from subcultured aliquots, to minimize subjective bias and enhance result validity.

### 2.4. MIC and MBC Determination

Minimum Inhibitory Concentration (MIC) and Minimum Bactericidal Concentration (MBC) of TiAB were assessed following the Clinical and Laboratory Standards Institute (CLSI) guidelines. MIC was defined as the lowest concentration of TiAB that visibly inhibited bacterial growth after 24 h of incubation at 35 ± 2 °C. To determine MBC, 10 µL aliquots were taken from the MIC well and three higher concentrations, then plated onto Columbia agar (COS) and incubated for 24 h at 37 °C. MBC was defined as the lowest concentration of TiAB resulting in ≥99.9% reduction in the initial viable count. Control wells were included in each assay: positive controls contained BHI broth with bacterial suspension, while negative controls contained only sterile BHI broth. Given the solid particulate nature and partial solubility of TiAB, MIC values are reported as % (*w*/*v*) suspensions. Direct conversion to standard mass units (μg/mL) is not applicable, in line with other studies on metal oxide nanomaterials. To better represent central tendencies across species, MIC and MBC values were expressed as medians, rather than means, due to the non-Gaussian distribution of the data and the presence of inter-strain variability. The use of medians provided a more robust measure of antimicrobial performance across clinical strains. Due to the partial solubility and visible sedimentation of TiAB in liquid medium, all MIC wells were gently vortexed for 5 s prior to visual inspection, to re-suspend the compound and allow clearer assessment of bacterial turbidity. Readings were performed consistently within 2 min post-vortexing to ensure standardization. In wells where turbidity was difficult to assess due to sediment masking, aliquots were subcultured on COS agar to confirm the MIC endpoint. This approach minimized variability and enhanced the reproducibility of MIC determinations.

### 2.5. Time-Kill Assay

The bactericidal kinetics of TiAB were evaluated using time-kill assays. Bacterial suspensions adjusted to 0.5 McFarland were incubated with TiAB at concentrations corresponding to 0.5× MIC, 1× MIC, and 2× MIC in a shaker–incubator at 37 °C with constant agitation, ensuring homogeneous exposure and limiting particle sedimentation. Aliquots were collected at 0, 2, 8, and 24 h, serially diluted, and plated on COS agar for viable count enumeration. Plates were incubated at 37 °C for 24 h, and colony-forming units per milliliter (CFU/mL) were determined. A ≥3 log_10_ CFU/mL reduction compared to the initial inoculum was interpreted as bactericidal activity; a reduction of <3 log_10_ CFU/mL was considered bacteriostatic. This approach allowed the characterization of TiAB’s antimicrobial dynamics over time and offered insights into its therapeutic potential for topical use in dermatological infections.

## 3. Results

**Figure 1:**
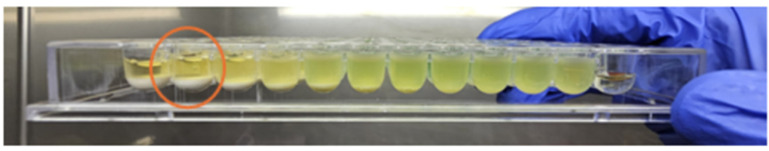
The figure shows an image of Pseudomonas aeruginosa strain 8 (highlighting its characteristic green hyperpigmentation). The MIC evaluations are circled in red, performed through visual observation in the first well where, above the powder deposit, the BHI solution appears clear like the negative control and without any signs of turbidity.

**Figure 2:**
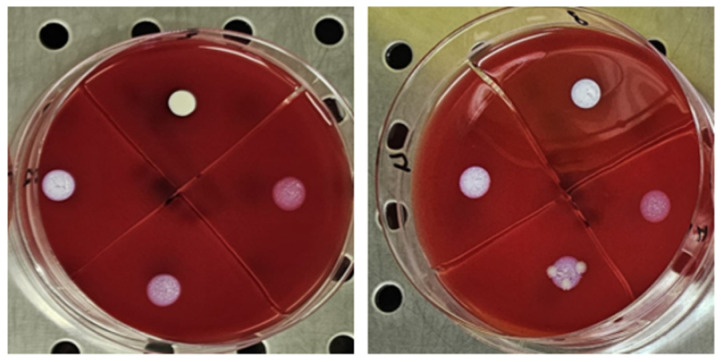
The figure shows the first 4 concentrations from the wells, plated on selective media in 10 µL for the MBC evaluation, from left to right for Pseudomonas aeruginosa and Klebsiella pneumoniae. The concentration that showed no growth was recorded.

The antimicrobial activity of TiAB against the tested clinical dermatological isolates is summarized in Table 1. MIC values represent the lowest TiAB concentration that inhibited visible bacterial growth after 24 h of incubation, while MBC values indicate the concentration required to achieve ≥99.9% reduction in viable bacterial counts on COS agar The complete dataset with MIC and MBC values for each individual strain is provided in Supplementary Table S1, offering insight into intra-species variability. Figure 1 illustrates representative results from MIC assays, with turbidity clearance observed above the sedimented TiAB indicating inhibition. MBC determinations were confirmed via subculture on agar plates (Figure 2).

Across all tested pathogens, TiAB showed consistent antimicrobial activity, with median MIC values ranging from 1% to 4%, and MBC values generally one or two dilution steps higher, confirming a concentration-dependent bactericidal effect. Gram-negative bacteria, including *Escherichia coli* and *Pseudomonas aeruginosa*, exhibited the greatest susceptibility (median MICs of 1% and 2%, respectively), with rapid bacterial clearance at low concentrations. Similarly, *Enterobacter cloacae*, *Klebsiella pneumoniae*, and ESBL-producing *E. coli* demonstrated median MIC values between 2% and 4%, supporting the efficacy of TiAB across a broad Gram-negative spectrum.

Gram-positive organisms showed more variable responses. *Streptococcus pyogenes*, *Staphylococcus aureus* (both MSSA and MRSA), and *Staphylococcus epidermidis* (MSSE and MRSE) exhibited higher MIC and MBC values overall, with median MICs of 4% and several undetermined MBC values (n/a) within the 24-h incubation. This suggests reduced bactericidal susceptibility within the tested concentration range.

To further explore this, selected Gram-positive strains with persistent turbidity at 24 h were incubated for an extended period of 48 h, and MIC values were reassessed. As shown in Table 2, a number of these strains demonstrated lower MICs after prolonged exposure (e.g., *S. pyogenes* and *S. epidermidis* MSSE showed inhibition at 4%). This delayed response suggests that TiAB exhibits time-dependent antimicrobial activity, potentially linked to gradual silver ion release from the TiO_2_ matrix in suspension. This behavior aligns with previous findings on metal-based powders such as silver, titanium, and zinc, known for their sustained antimicrobial activity [20].

Overall, TiAB demonstrated a broad-spectrum antimicrobial profile. While rapid activity was seen in Gram-negative isolates, Gram-positive pathogens may require higher concentrations or prolonged contact time to achieve comparable inhibition. These findings support the potential application of TiAB in dermatological infections, particularly those associated with chronic wounds where sustained topical antimicrobial activity is desired.

### Time-Kill Assay Results

**Figure 3:**
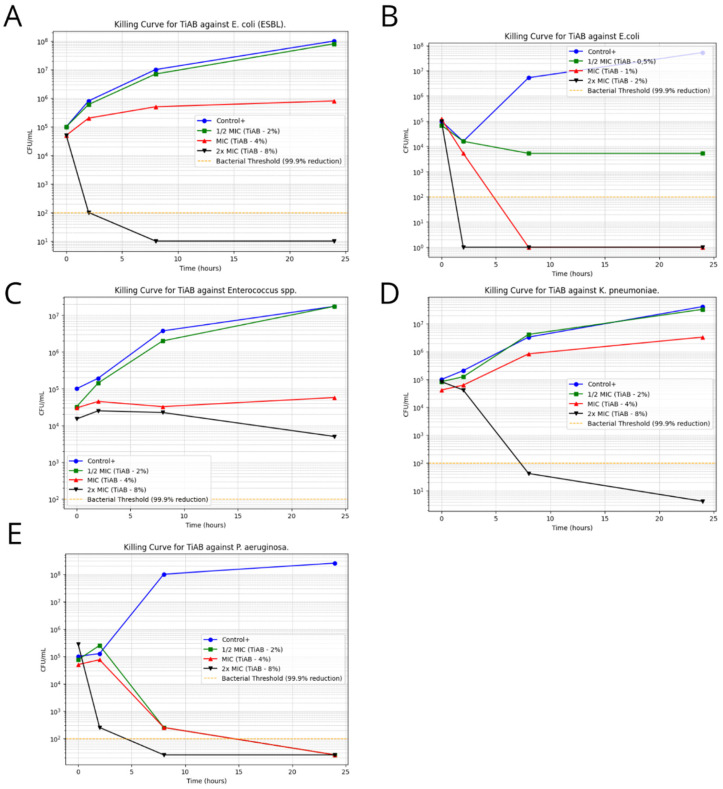
Time-Kill Curves for TiAB Against Clinical Isolates. Bacterial survival curves illustrating the antimicrobial effect of TiAB at 0.5× MIC, 1× MIC, and 2× MIC concentrations over 24 h. A ≥3 log_10_ CFU/mL reduction was considered indicative of bactericidal activity. Rapid and complete eradication was achieved for *E. coli* and *K. pneumoniae* at 2× MIC within 4–8 h (**A**,**B**,**D**). *Pseudomonas aeruginosa* also showed a strong response, with full bacterial clearance by 6 h at 2× MIC, while 1× MIC caused delayed growth without complete inhibition (**E**). *Enterococcus* spp. exhibited a slower, time-dependent decline in CFU/mL at 2× MIC (**C**), without full bactericidal effect within 24 h. No significant killing was observed in Gram-positive bacteria at tested MIC levels, highlighting the need for extended exposure or higher concentrations to achieve comparable efficacy.

Time-kill assays were performed on representative clinical isolates to assess the temporal bactericidal activity of TiAB at 0.5×, 1×, and 2× MIC concentrations. The results revealed a clear concentration- and time-dependent effect, particularly against Gram-negative organisms. Figure 3 illustrates the bacterial growth kinetics for selected strains.

*Escherichia coli* demonstrated a rapid decline in viability, with complete eradication observed within 4 h at 2× MIC (2%), confirming its high susceptibility to TiAB. Similarly, ESBL-producing *E. coli* required slightly prolonged exposure, with significant CFU/mL reductions noted by 6 h and near-total killing by 8 h. This delay may be attributed to the presence of resistance mechanisms, such as β-lactamase activity, that may partially influence TiAB efficacy.

*Klebsiella pneumoniae* and *Pseudomonas aeruginosa* also responded strongly to TiAB. At 2× MIC, both species exhibited ≥3 log_10_ CFU/mL reductions within 6 to 8 h, with *P. aeruginosa* showing near-complete bacterial killing despite its known intrinsic resistance via efflux pumps and biofilm formation. These results support the hypothesis that TiAB maintains potent bactericidal activity even against difficult-to-treat Gram-negative pathogens.

In contrast, Gram-positive bacteria showed a less consistent response. *Enterococcus* spp., tested at 2× MIC (8%), displayed a gradual bactericidal effect, with a reduction close to ≥3 log_10_ observed by 24 h, but without full eradication. This suggests that TiAB exerts a slower killing dynamic in certain Gram-positive organisms, likely requiring prolonged contact to reach maximal efficacy.

For other Gram-positive strains, including *Streptococcus pyogenes*, *Staphylococcus aureus* (both MSSA and MRSA), and *Staphylococcus epidermidis* (MSSE and MRSE), time-kill assays were not performed due to undetermined or ≥8% MBC values, which exceeded the upper limits of the tested TiAB concentrations. Additionally, considering the results of MIC determinations at 48 h, a longer exposure period would likely be necessary to fully assess bactericidal kinetics in these species.

Overall, these findings reinforce the rapid and potent antimicrobial action of TiAB against Gram-negative bacteria, particularly *E. coli*, *K. pneumoniae*, and *P. aeruginosa*. Meanwhile, the activity against Gram-positive bacteria appears to be slower and concentration-dependent, supporting the hypothesis of a gradual ion release mechanism and suggesting that extended exposure times or higher concentrations may be necessary to achieve complete bacterial clearance in this group.

## 4. Discussion

### 4.1. Interpretation of Results and Relevance to Antimicrobial Applications

This study provides important insights into the in vitro efficacy of TiAB, a silver-ion bound titanium dioxide compound, against a wide range of clinical bacterial isolates commonly associated with dermatological infections [19]. The findings demonstrate that TiAB exerts a strong, rapid bactericidal effect against Gram-negative pathogens such as *Escherichia coli*, *Klebsiella pneumoniae*, *Enterobacter cloacae*, and *Pseudomonas aeruginosa*. These bacteria showed low MIC and MBC median values (generally ≤2% and ≤4%, respectively), with time-kill curves confirming complete eradication at 2× MIC within 4–8 h.

Conversely, Gram-positive bacteria—including *Streptococcus pyogenes*, *Staphylococcus aureus* (MSSA and MRSA), and *Staphylococcus epidermidis* (MSSE and MRSE)—exhibited higher MIC values, often ≥8%, and undetermined MBC values within the 24 h timeframe. For these species, no significant bactericidal effect was observed during the time-kill assay [17]. Given this outcome and the known time-dependent action of certain metal-based antimicrobials, an extended MIC incubation at 48 h was conducted for representative Gram-positive strains. Notably, this prolonged exposure resulted in decreased MIC values (e.g., from ≥8% to 4% for *S. pyogenes* and MSSA), confirming that TiAB maintains bacteriostatic and potentially bactericidal activity over extended periods.

Among Gram-positive bacteria, *Enterococcus* spp. represented an intermediate profile, with 24 h MIC and MBC values higher than those of Gram-negative strains, but with observable bactericidal activity at 2× MIC over 24 h. This suggests that TiAB may be effective in treating infections caused by *Enterococcus*, potentially including vancomycin-resistant strains (VRE), especially when prolonged exposure is feasible [14,15].

These findings support the potential of TiAB for dermatological use, particularly in chronic wound settings where long-term topical application is possible. The delayed but sustained effect observed against Gram-positive pathogens may be advantageous in formulations designed for slow release or continuous contact.

### 4.2. Differential Response Between Gram-Negative and Gram-Positive Bacteria

A key observation emerging from this study is the differential response of Gram-negative and Gram-positive bacteria to TiAB. The higher susceptibility of Gram-negative bacteria is likely related to their cell envelope structure, which, although possessing an outer membrane, contains porins that facilitate the diffusion of silver ions. These ions can reach their intracellular targets more efficiently, enabling rapid bactericidal action.

In contrast, Gram-positive bacteria possess a thick peptidoglycan layer and lack an outer membrane. This structural difference may reduce the permeability to silver ions, thereby slowing TiAB’s antimicrobial effect. Additionally, Gram-positive species frequently engage in biofilm formation, which acts as a physical barrier to antimicrobial penetration. Biofilm-associated resistance mechanisms, including efflux pumps and extracellular matrix components, could further reduce TiAB efficacy in the short term.

Emerging studies on silver nanoparticles (AgNPs) suggest that *flagellin*, a protein secreted by Gram-negative bacteria, may aggregate AgNPs and enhance their reactivity at the bacterial surface [11]. Although TiAB differs structurally, it is possible that similar mechanisms facilitate its stronger action on Gram-negative species. Conversely, Gram-positive bacteria do not express flagellin and may thus fail to enhance silver-ion reactivity at the same rate.

The observed time-dependent MIC decrease at 48 h supports previous literature indicating that the antimicrobial efficacy of metal-based compounds—especially those with complex crystalline matrices like TiAB—may increase over time. This justifies clinical strategies involving prolonged topical exposure, such as wound dressings, creams, or impregnated gauzes [12,18].

This study has some limitations. The data are based exclusively on in vitro assays; in vivo confirmation in animal wound models is required to assess safety, pharmacokinetics, and true therapeutic potential. Cytotoxicity on keratinocytes or fibroblasts was not evaluated and should be addressed before clinical application. Additionally, the use of visual turbidity readings may introduce minor subjectivity despite colony count confirmation. These aspects will be addressed in future studies.

### 4.3. Safety Profile and Dermatological Considerations

Although the present study confirms the antimicrobial efficacy of TiAB against a wide range of pathogens, its clinical application—particularly in dermatology—must also consider safety and tolerability, especially on compromised skin. Silver and titanium dioxide nanomaterials are increasingly used in biomedical fields due to their broad-spectrum antimicrobial activity and potential for integration into wound care devices. Recent studies have highlighted that silver-doped TiO_2_ systems can exert bactericidal effects against pathogens such as *S. aureus* and *E. coli*, without inducing significant cytotoxicity in vitro [10,21,22]. These nanomaterials act through multiple mechanisms, including the generation of reactive oxygen species (ROS) and targeted disruption of bacterial membranes via electrostatic interactions [23,24,25]. Notably, silver nanoparticles have already been investigated in the context of wound dressings for chronic ulcers and have shown favorable results in limiting colonization by *P. aeruginosa* and *S. aureus* [26]. Nevertheless, while TiAB shares similar compositional characteristics, we recognize that specific cytotoxicity testing on skin-derived cell lines (e.g., keratinocytes and fibroblasts) is essential to assess its compatibility with human tissue. Therefore, further studies will be needed to evaluate TiAB’s dermatological safety profile before advancing toward clinical translation.

## 5. Conclusions

This study confirms that TiAB exerts robust in vitro antimicrobial activity against a wide spectrum of Gram-negative bacteria commonly implicated in dermatological infections, including *Escherichia coli*, ESBL-producing *E. coli*, *Klebsiella pneumoniae*, *Enterobacter cloacae*, and *Pseudomonas aeruginosa*. These pathogens exhibited low MIC and MBC values, with complete bacterial clearance observed at 2× MIC within 4 to 8 h—highlighting TiAB’s potential as a fast-acting topical agent, particularly in cases involving antibiotic-resistant strains. In contrast, Gram-positive bacteria—including *Staphylococcus aureus* (MSSA and MRSA), *Streptococcus pyogenes*, and *Staphylococcus epidermidis*—demonstrated reduced susceptibility in the same timeframe, with MIC values ≥8% and no detectable bactericidal activity at tested concentrations during the initial 24 h. However, extended incubation up to 48 h revealed a time-dependent decrease in MIC values for several Gram-positive isolates, suggesting that TiAB retains antimicrobial potential against these species when exposure is prolonged. Taken together, these results reinforce TiAB’s suitability for the treatment of superficial and chronic skin infections caused by Gram-negative pathogens, particularly in wound care settings where sustained local exposure can be achieved. For Gram-positive pathogens, further optimization strategies—including prolonged application, dose adjustment, or synergistic combination with other antimicrobials—should be explored to maximize efficacy.

Future directions should include in vivo studies to evaluate TiAB’s safety, pharmacodynamics, and therapeutic potential in clinical dermatological applications. Investigating its use in biofilm-related infections, optimizing formulation for sustained release, and assessing synergy with conventional antibiotics could further support TiAB’s development as a novel topical antimicrobial agent.

In conclusion, while TiAB shows promising in vitro antimicrobial and antibiofilm activity, comprehensive in vivo studies are crucial to confirm its biocompatibility and effectiveness as a topical agent for dermatological infections.

## Figures and Tables

**Table 1:** Median MIC and MBC/MFC values of TiAB against clinically relevant vaginal pathogens.

Bacterial Species	No. of Isolates	MIC Median (% *w*/*v*)	MBC Median (% *w*/*v*)	IQR (MIC) (% *w*/*v*)
*Staphylococcus aureus* (MSSA)	15	2.0	4.0	1.5–2.0
*Staphylococcus aureus* (MRSA)	15	2.0	4.0	2.0–4.0
*Staphylococcus epidermidis* (MSSE)	10	2.0	4.0	2.0–4.0
*Staphylococcus epidermidis* (MRSE)	10	2.0	4.0	2.0–4.0
*Streptococcus pyogenes*	15	2.0	4.0	1.0–2.0
*Escherichia coli*	15	1.0	2.0	0.5–1.0
*Escherichia coli* (ESBL+)	15	1.0	2.0	0.5–1.0
*Klebsiella pneumoniae*	15	1.0	2.0	0.5–1.0
*Enterobacter cloacae*	15	1.0	2.0	0.5–1.0
*Enterococcus* spp.	15	2.0	4.0	1.5–2.0
*Pseudomonas aeruginosa*	15	1.0	2.0	0.5–1.0

MIC and MBC values are expressed as % (*w*/*v*) TiAB suspensions. Due to the hybrid structure and limited solubility, direct conversion to standard μg/mL units is not feasible. Values represent median (IQR) for each bacterial species tested.

**Table 2:** MIC values extended at 48 h.

Pathogen	Strahin	MIC (% *w*/*v*) 48 h
*Streptococcus pyogenes*	11	4%
*Staphylococcus aureus* (MSSA)	4	4%
*Staphylococcus aureus* (MRSA)	3	8%
*Staphylococcus epidermidis* (MSSE)	6	4%
*Staphylococcus epidermidis* (MRSE)	8	8%
*Enterococcus* spp.	6	1%
	7	0.5%

Minimum inhibitory concentration (MIC) values were reassessed after 48 h in strains showing no clear inhibition at 24 h. The extended exposure allowed the evaluation of time-dependent antimicrobial effects of TiAB, particularly for Gram-positive bacteria in which bacteriostatic activity was suspected. These data suggest a progressive, delayed inhibition possibly related to sustained ion release from TiAB sediment in broth.

## Data Availability

The data that support the findings of this study are not openly available due to reasons of sensitivity and are available from the corresponding author upon reasonable request. Data are stored in a controlled access repository at IRCCS MultiMedica (Milan, Italy).

## Supplementary Materials

The following supporting information can be downloaded at: https://www.mdpi.com/article/10.3390/diseases13090277/s1, Table S1: MIC and MBC values of TiAB against clinical total isolates.

## Abbreviations

MIC	Minimum Inhibitory Concentration
MBC	Minimum Bactericidal Concentration
CFU	Colony Forming Units
TiAB	Titanium dioxide microcrystals with silver ions
CLSI	Clinical and Laboratory Standards Institute
