# Supplementary material for: In Vitro Activity of Silver-Bound Titanium Dioxide (Tiab) Against Multidrug-Resistant Bacteria from Dermatological Infections
Source: Diseases. 2025 Aug 22;13(9):277. doi: 10.3390/diseases13090277 (PMC12468273; doi:10.3390/diseases13090277)
Supplement: Supplementary file 1 [file diseases-13-00277-s001.zip › diseases-3764991-supplementary.pdf]

Table S1. MIC and MBC values of TiAB against clinical total isolates

Pathogen	Strain	MIC	MIC range	MBC	MBC range
Streptococcus pyogenes	1	2%	**1% < MIC < 8%**	4%	**4% < MBC < 8%**
	2	8%		n/a	
	3	1%		4%	
	4	4%		8%	
	5	4%		8%	
	6	1%		4%	
	7	8%		n/a	
	8	1%		4%	
	9	4%		4%	
	10	4%		4%	
	11	n/a		n/a	
	12	4%		4%	
	13	2%		8%	
	14	4%		8%	
	15	1%		4%	
Staphylococcus aureus (MSSA)	1	4%	**2% < MIC < 8%**	n/a	**MBC ≥ 8%**
	2	n/a		n/a	
	3	8%		n/a	
	4	n/a		n/a	
	5	4%		8%	
	6	4%		8%	
	7	4%		8%	
	8	4%		8%	
	9	4%		8%	
	10	2%		8%	
	11	4%		8%	
	12	4%		n/a	
	13	4%		8%	
	14	2%		8%	
	15	8%		n/a	
Staphylococcus aureus (MRSA)	1	2%	**2% < MIC < 8%**	4%	**4% < MBC< 8%**
	2	4%		8%	
	3	n/a		n/a	
	4	4%		8%	
	5	4%		n/a	
	6	4%		8%	
	7	2%		4%	
	8	4%		8%	
	9	2%		4%	
	10	4%		8%	
	11	8%		n/a	
	12	2%		4%	
	13	4%		8%	
	14	4%		8%	
	15	n/a		n/a	
Staphylococcus epidermidis (MSSE)	1	4%	**2% < MIC < 8%**	8%	**4% < MBC < 8%**
	2	4%		8%	
	3	4%		n/a	
	4	4%		8%	
	5	2%		4%	
	6	n/a		n/a	
	7	2%		4%	
	8	4%		8%	
	9	2%		4%	
	10	4%		8%	
Staphylococcus epidermidis (MRSE)	1	1%	**1% < MIC < 8%**	8%	**2% < MBC < 8%**

	2	4%		8%	
	3	8%		n/a	
	4	8%		n/a	
	5	1%		4%	
	6	2%		8%	
	7	4%		8%	
	8	n/a		n/a	
	9	4%		8%	
	10	4%		8%	
Escherichia coli	1	1%	**0,5% < MIC < 2%**	4%	**1% < MBC < 4%**
	2	1%		4%	
	3	0,5%		1%	
	4	1%		2%	
	5	2%		2%	
	6	1%		2%	
	7	0,5%		4%	
	8	1%		4%	
	9	1%		2%	
	10	2%		4%	
	11	0,5%		1%	
	12	1%		2%	
	13	1%		2%	
	14	2%		4%	
	15	1%		2%	
Enterobacter cloacae	1	2%	**1% < MIC < 4%**	8%	**4% < MBC < 8%**
	2	2%		n/a	
	3	1%		4%	
	4	2%		8%	
	5	4%		n/a	
	6	4%		8%	
	7	4%		n/a	
	8	4%		n/a	
	9	2%		4%	
	10	2%		8%	
	11	4%		n/a	
	12	2%		8%	
	13	2%		4%	
	14	1%		4%	
	15	4%		n/a	
Klebsiella pneumomae	1	1%	**1% < MIC < 8%**	1%	**MBC > 8%**
	2	8%		8%	
	3	8%		8%	
	4	4%		4%	
	5	4%		4%	
	6	8%		8%	
	7	8%		8%	
	8	8%		8%	
	9	1%		2%	
	10	4%		8%	
	11	4%		8%	
	12	8%		8%	
	13	8%		n/a	
	14	4%		8%	
	15	4%		8%	
Escherichia coli (ESBL)	1	8%	**2% < MIC < 8%**	n/a	**4% < MBC < 8%**
	2	2%		4%	
	3	4%		8%	
	4	4%		8%	

	5	4%		8%	
	6	4%		8%	
	7	4%		8%	
	8	8%		8%	
	9	4%		8%	
	10	4%		8%	
	11	4%		8%	
	12	2%		4%	
	13	2%		4%	
	14	4%		8%	
	15	4%		8%	
Enterococcus spp.	1	4%	**1% < MIC < 4%**	n/a	**4% < MBC < 8%**
	2	4%		8%	
	3	2%		8%	
	4	4%		8%	
	5	2%		8%	
	6	4%		8%	
	7	4%		8%	
	8	2%		4%	
	9	4%		8%	
	10	2%		8%	
	11	1%		4%	
	12	1%		8%	
	13	2%		4%	
	14	4%		8%	
	15	1%		4%	
Pseudomonas aeruginosa	1	4%	**1% < MIC < 8%**	4%	**2% < MBC < 8%**
	2	2%		8%	
	3	2%		2%	
	4	1%		1%	
	5	4%		4%	
	6	4%		4%	
	7	2%		2%	
	8	8%		8%	
	9	2%		4%	
	10	2%		4%	
	11	4%		8%	
	12	1%		8%	
	13	4%		8%	
	14	4%		>8%	
	15	2%		4%	

Complete MIC and MBC values of TiAB for all 155 clinical isolates tested in this study. Each row reports the MIC and MBC values for a single strain, grouped by pathogen species. On the right, the median MIC and MBC values for each species are expressed as percentages (%). These data provide a detailed overview of the inter-strain variability in TiAB susceptibility among pathogens.
